# Two cases of neurilemmoma in the nasal vestibule: A case report and literature review

**DOI:** 10.1097/MD.0000000000029006

**Published:** 2022-03-11

**Authors:** Sung Jae Heo, A. Young Kim, Jung Soo Kim

**Affiliations:** Department of Otorhinolaryngology-Head and Neck Surgery, School of Medicine, Kyungpook National University, Daegu, South Korea.

**Keywords:** neoplasm, neurilemmoma, nose

## Abstract

**Rationale:**

Neurilemmoma is a benign tumor derived from the Schwann cells of the nerve sheath. The highest incidence of neurilemmoma occurs in the head and neck region; however, the nose and paranasal sinuses are rarely involved. Less than 4% of these tumors involve the nasal cavity and paranasal sinuses. To date, only six cases of nasal vestibule neurilemmoma have been reported.

**Patient concerns:**

Two patients (a 32-year-old man and a 42-year-old woman) visited our clinic with complaint of a lump in the left nasal vestibule.

**Diagnosis:**

Histopathological examination and immunohistochemical staining confirmed a neurilemmoma.

**Interventions:**

The mass was completely removed via an intranasal approach.

**Outcomes and Lessons:**

Neurilemmoma is easy to overlook because it occurs rarely in the nasal vestibule, but neurilemmoma needs to be considered as a differential diagnosis.

## 1 Introduction

Neurilemmoma is a benign tumor derived from Schwann cells of the nerve sheath and can occur in all peripheral nerves, excluding the optic and olfactory nerves, the sympathetic nerves, and the cranial nerves. Neurilemmoma occurs predominantly in the head and neck region, and the incidence of tumors involving the nasal cavity or sinuses is rare, with only 4% of all cases.^[1]^ Of these, neurilemmoma of the nasal vestibule is extremely rare, and only six cases have been reported nationally to date.^[2–7]^ We experienced two cases of neurilemmoma in the left nasal vestibule in a 32-year-old male patient and a 42-year-old female.

## 2 Case report

### 2.1 Case 1

**Figure 1:**
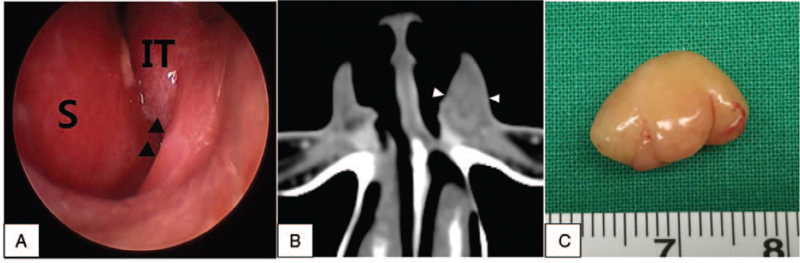
A bulging of the left vestibule is shown in the nasal endoscope (A). Computed tomography shows a mass in the left vestibule (B). The tumor is a 1.5 cm-sized yellowish mass with a smooth surface (C). IT = inferior turbinate, S = nasal septum.

A 32-year-old male patient with a painless tumor, growing for several years, in the left nasal vestibule was admitted. Nasoendoscopy showed a protruding left nasal vestibule (Fig. 1A). Tenderness of the region was not noted; however, a soft and tense tumor was palpated. Computed tomography (CT) revealed a 1.5 cm clearly demarcated soft tissue shadow in the left nasal vestibule region (Fig. 1B), and no changes in the nearby tissues or bone destruction were observed.

**Figure 2:**
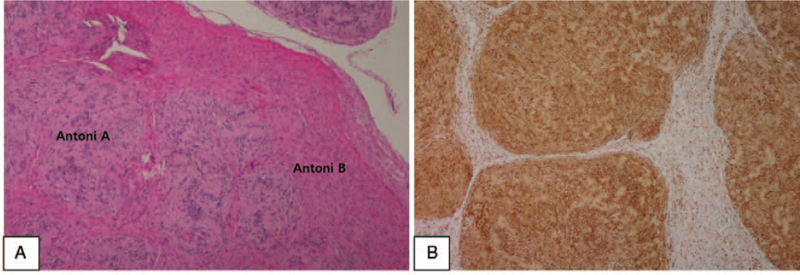
H&E staining of the tumor shows Antoni A and B patterns which are remarkable features of neurilemmoma (A, ×100). Immunohistochemical staining for S-100 protein demonstrates positive immunoreactivity in spindle-shaped tumor cells (B, ×100).

A significantly deviated septum on the left side was observed, and therefore, the excision of the tumor of the nasal vestibule and septoplasty were performed simultaneously under general anesthesia. An incision of 1.5 cm from inside the left ala to the nasal vestibule was generated. Next, the nearby soft tissues were peeled off, and the oval-shaped tumor measuring 1.5 cm × 1 cm, covered with a smooth film, was detected and removed (Fig. 1C). Pathological evaluation of the excised tumor using hematoxylin and eosin staining showed repeated Antoni A type patterns, which were aggregated fusiform cells, and Antoni B type patterns, which had fewer cells. S-100 immunostaining was positive, and therefore, neurilemmoma was confirmed (Fig. 2). Postoperative bleeding, infection, adhesion, or complications were absent. The patient was followed up for 2 years and 6 months and presented no recurrence.

### 2.2 Case 2

**Figure 3:**
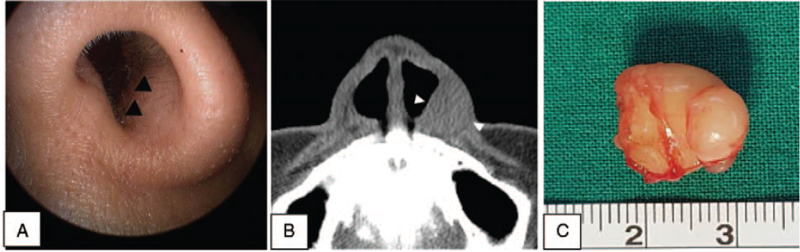
A protrusion of the left vestibule is observed in the endoscopic examination (A). Computed tomography shows a relatively well-circumscribed ovoid mass in the left nasal vestibule (B). A gross picture of the mass shows a 1.5 cm-sized yellowish tumor with a smooth surface (C).

**Figure 4:**
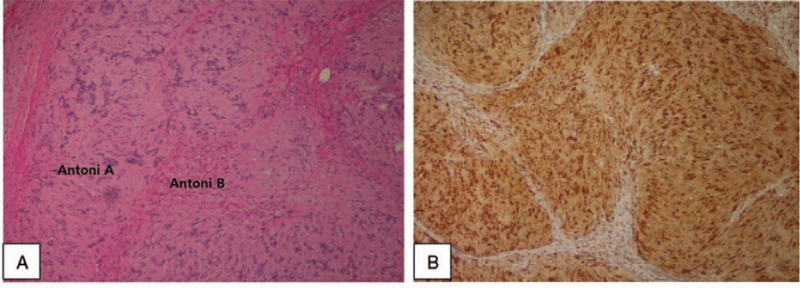
Histology of the tumor shows Antoni A and B patterns in the H&E staining (A, ×100) and immunoreactivity for S-100 protein (B, ×100).

A 42-year-old patient with a tumor, growing since the past year, in the left nasal vestibule was admitted. No pain or tenderness in the area was noted; however, she complained of left nasal congestion. A tumor protruding into the nasal cavity of the left nasal vestibule was observed using nasoendoscopy (Fig. 3A). It was firm on palpation, and CT showed a 1.5 cm homogenous shadow in the left nasal vestibule. Calcification, cystic degeneration, or destruction of the nearby bones around the tumor were not observed (Fig. 3B). A 2-cm marginal incision from the left alar rim to the nasal floor was generated by approaching through the intranasal space. Surrounding tissues were peeled, and subsequently, the tumor measuring 1.5 cm × 1.5 cm, covered with a smooth film, was re-moved (Fig. 3C). Neurilemmoma was diagnosed from the histopathologic evaluation (Fig. 4), and postoperative complications, such as infection or bleeding, were absent. The patient was followed up for 2 years and presented no recurrence.

## 3 Discussion

Neurilemmoma grows gradually with no accompanying subjective symptoms at the early phase; however, with tumor growth, pain appears due to pressure in the originating nerves.^[8]^ Tumors in the nasal cavity are generally associated with the most common symptom of nasal congestion around the lesion; other symptoms, such as pain, headache, or anosmia, may manifest with tumor growth.^[9]^ Based on the analysis of the clinical presentation of neurilemmoma in the nasal vestibule according to the Korean literature, most patients reported mild nasal congestion or presented no symptoms, except a few cases with pain or nasal bleeding (Table 1). However, neurilemmoma in the nasal septum is frequently associated with unilateral nasal bleeding, severe nasal congestion, and headaches.^[6,8,9]^ The severity of symptoms varies depending on the location of the tumor; for example, a tumor in the nasal vestibule can be easily identified by the patients in their early phase, and the disease may be diagnosed before symptom appearance due to the growing tumor.

**Table 1:** Summary of the neurilemmoma cases involving nasal vestibule.

Reference	Sex/age	Right/left	Size (cm)	Symptom	CT finding	Surgical approach	Anesthesia	F/U (mo)	Recurrence
Yoo and Yun^[2]^	F/51	Left	1.4	Palpable mass nasal obstruction	ND	Simple excision	ND	ND	ND
Ko et al^[4]^	F/47	Left	0.7	Palpable mass	ND	Simple excision	ND	ND	No
Boo et al^[5]^	M/48	Left	2	Palpable mass nasal obstruction epistaxis (−)	Well defined bone destruction (−)	Sublabial incision	G/A	6	No
Hu et al^[6]^	M/59	Right	2	Nasal obstruction	Well defined uneven density mild enhancement	Gingivobuccal incision	L/A	152	No
Hu et al^[6]^	F/27	Left	1.8	Nasal obstruction	Well defined patchy enhancement	Gingivobuccal incision	L/A	147	No
Zizhen et al^[7]^	ND	ND	ND	ND	Well defined uneven density slightly enhancement bone of the adjacent maxillary sinus	ND	ND	ND	No
Present case 1	M/32	Left	1.5	Palpable mass	Well defined uneven density cystic change (−) bone destruction (−)	Marginal incision	G/A	20	No
Present case 2	F/42	Left	1.5	Palpable mass nasal obstruction	Well defined cystic change (−) bone destruction (−)	Marginal incision	G/A	7	No

CT = computed tomography, F/U = follow-up, G/A = general anesthesia, L/A = local anesthesia, ND = no data.

Neurilemmoma generally appears as contrast-enhanced homogenous soft tissue shadow on the CT image; however, in necrotic cases or cystic degeneration, heterogeneous shadow or calcification may be observed. Further, large-size tumors can cause bone destruction or deformation due to pressure.^[7]^ In this study, CT of neurilemmoma in the nasal vestibule showed a clear demarcation from the nearby areas, and the shadows of the inside were mostly heterogeneous similar to other reports from Korean literature. Destruction of nearby bones was observed in only one case. Bone destruction may occur in the case of large tumor size; however, both cases in this report presented a small tumor size <2 cm, which may explain the absence of bone destruction.

Surgical removal is the best treatment option for neurilemmoma.^[7]^ During the excision, the affected nerves should be handled with care. Moreover, incomplete excisions are associated with a risk of recurrence. The recurrence rate is rare when tumors are completely excised. Therefore, a wide range of excision to the nearby tissues is unnecessary. Cases in this report did not have adhesion to the nearby tissues during the excision of tumors, and hence, the tumors were easily removed. Analysis of these two cases and other reported national and international cases suggests that pedunculated tumors might simply be excised. Additionally, the excision of a tumor on the mucous membrane requires minimally invasive surgery through the gum or a nasal incision. In contrast, surgical cases through the left nasal incision for neurilemmoma in the nasal septum or sinus have been reported^[6,9,10]^; tumors in the nasal vestibule diagnosed early entails a surgical benefit since the disease is exposed and can be easily detected.

Neurilemmoma in the nasal cavity is thought to originate from the ophthalmic branch, maxillary branch, or autonomic ganglion of the trigeminal nerve.^[3]^ Cases in this report included tumors in the lower part of the nasal vestibule; thus, the tumors might have originated from the lower ophthalmic branch of the maxillary branch in the trigeminal nerves or unbranched area. The nerve branch, thought to be the origin, was not identified during surgery, and there were no postoperative functional disabilities or sensory disabilities.

For the excision of neurilemmoma in the nasal vestibule, most cases have reported surgery using local anesthesia, and only one case has reported general anesthesia for the surgery along with obstructive sleep apnea. In case 1 of this report, general anesthesia was performed for the simultaneous fixation of the nasal septum. In case 2, general anesthesia was performed based on the patient's preference due to a history of panic disorder. However, based on the size and the location of the tumor, local anesthesia would have been sufficient.

Neurilemmoma is typically not malignant, but reports of transition into a malignant form after a long-term follow-up without surgical removal are available.^[11,12]^ Therefore, surgical removal should be performed for treatment and accurate diagnosis, and a follow-up period is needed to confirm the recurrence of residual tumor in case of incomplete excision.

## Author contributions

**Conceptualization:** Sung Jae Heo.

**Data curation:** Sung Jae Heo, A Young Kim, Jung Soo Kim.

**Formal analysis:** Sung Jae Heo, A Young Kim, Jung Soo Kim.

**Investigation:** Sung Jae Heo, A Young Kim, Jung Soo Kim.

**Methodology:** Sung Jae Heo, A Young Kim, Jung Soo Kim.

**Project administration:** Sung Jae Heo.

**Supervision:** Sung Jae Heo, Jung Soo Kim.

**Writing – original draft:** Sung Jae Heo, A Young Kim.

**Writing – review & editing:** Sung Jae Heo, Jung Soo Kim.
